# Testing Equality of Multiple Population Means under Contaminated Normal Model Using the Density Power Divergence

**DOI:** 10.3390/e24091189

**Published:** 2022-08-25

**Authors:** Jagannath Das, Beste Hamiye Beyaztas, Maxwell Kwesi Mac-Ocloo, Arunabha Majumdar, Abhijit Mandal

**Affiliations:** 1 Department of Mathematical Sciences, University of Texas at El Paso, El Paso, TX 79968, USA; 2 Department of Statistics, Istanbul Medeniyet University, Istanbul 34700, Turkey; 3 Department of Mathematics, Indian Institute of Technology Hyderabad, Kandi 502284, Telangana, India

**Keywords:** minimum density power divergence, robust ANOVA, fixed effects, robust testing, M-estimation

## Abstract

This paper considers the problem of comparing several means under the one-way Analysis of Variance (ANOVA) setup. In ANOVA, outliers and heavy-tailed error distribution can seriously hinder the treatment effect, leading to false positive or false negative test results. We propose a robust test of ANOVA using an M-estimator based on the density power divergence. Compared with the existing robust and non-robust approaches, the proposed testing procedure is less affected by data contamination and improves the analysis. The asymptotic properties of the proposed test are derived under some regularity conditions. The finite-sample performance of the proposed test is examined via a series of Monte-Carlo experiments and two empirical data examples—bone marrow transplant dataset and glucose level dataset. The results produced by the proposed testing procedure are favorably compared with the classical ANOVA and robust tests based on Huber’s M-estimator and Tukey’s MM-estimator.

## 1. Introduction

The analysis of variance (ANOVA) has become one of the most useful and powerful statistical approaches in diverse applications, such as biology, physics, chemistry, genetics, engineering, economics, psychology, and medicine. This omnibus procedure, developed by [1], has often been applied to the continuous data from more than two independent samples for exploratory and confirmatory data analysis (cf. [2]). ANOVA is statistically appealing since this approach specifies and quantifies the effect of different treatments on the observed outcomes by comparing two sources of variabilities, i.e., variations within and between groups, to assess the equality of group means (or to test the null hypothesis of no treatment effects) (cf. [3,4]).

The classical ANOVA test requires some restrictive assumptions, such as normality of the errors, homogeneity of group variances, and absence of outliers, which may not be satisfied in practice (cf. [3,5,6,7]). In particular, one crucial issue that requires special attention is the presence of outliers that differ from the bulk of the data (cf. [8]). Outliers caused by the measurement error, recording error, and naturally atypical observations may be masked and have adverse effects on the efficiency of traditional estimators [8]. As a result, even a small deviation from the ideal conditions can make the test meaningless and lead to unreliable results. To downplay this problem, practitioners often use some ad-hoc methods to remove outliers. However, such an approach has shortcomings as it can lead to a significant loss of efficiency. Hence, a more appropriate strategy is to use a robust procedure that is not influenced by outlying observations but correctly describes the structure of the bulk of the data.

The research on the robustness of testing procedures starts with the study of [9] that explores the non-robustness of the classical ANOVA. Ref. [10] presents some key concepts related to robustness, such as breakdown point, influence function, the robustness of validity, and robustness of efficiency. The robustness of validity and efficiency is the two-fold purpose of performing robust testing. These concepts refer to the stability of the level of the test and maintaining the good power under the arbitrary departures from the null hypothesis and alternative hypothesis, respectively [11]. The robust procedures can be viewed as methods to check the appropriateness of the classical techniques. The readers are referred to [12,13,14,15] for more information about the robustified tests and their advantages over the classical testing procedures. Several procedures have been proposed to perform the ANOVA test robustly. For instance, refs. [16,17] proposed several robust methods for testing treatment effects using M-estimators. In addition, by adapting the M-estimates to the hypothesis tests in linear models, ref. [18] developed the likelihood ratio type tests to provide robust ANOVA. Moreover, ref. [19] examined the properties of $F^{*}$ and *W* statistics proposed by [20] replacing the usual ANOVA *F* test in the presence of heterogeneous variances. Using the median and trimmed mean, a robust one-way ANOVA under possibly non-regular conditions was proposed by [21]. More recently, ref. [22] proposed a robust test based on a weighted sum of squares for the one-way ANOVA model when the homoscedasticity is violated. Some non-parametric procedures are discussed in [10,23,24]. By combining the results of some existing studies related to non-parametric analysis, ref. [23] generalized the ANOVA model by relaxing the normality assumption as well as the structure of the designs. The authors used linear rank statistics to make statistical inferences about the treatment effects in obtaining a unified approach for continuous and discontinuous distribution functions. Ref. [24] suggested employing rank-based methods in the ANOVA test when there is a concern related to the presence of outliers in the data since the classical ANOVA methods may be conservative. The non-parametric methods are good choices when one might suspect the normality assumption in some practical cases. However, robust methods are generally preferred over the non-parametric (rank-based) ones because those methods produce stable results with a small loss of efficiency by allowing for limited changes in the data or small departures from the model assumptions (cf. [25,26,27,28]). Hence, we focus on the robust tests in this study because they are more generally powerful than the non-parametric tests and insensitive to violating some assumptions. In addition to the methodological studies, the ANOVA test is one of the most commonly applied methods in practical problems. Consult [28,29,30,31,32,33], and the references therein for a comprehensive review of the applications of robust ANOVA methods in medical, clinical, and pharmaceutical studies.

In this paper, we propose a robust test based on the minimum density power divergence estimator (MDPDE) introduced by [34] under weaker conditions. Based on the MDPDE, a one-sample Wald-type test is proposed in [35], and [36] develops a test for the equality of two sample means. These tests have substantially superior performance compared to the likelihood-based test in the presence of outliers, although being very competitive in pure data. So, the tests based on the MDPDE are very useful practical tools in robust statistics (cf. [37,38]).

The rest of the paper is organized as follows. First, we introduce the generalized analysis of variance model in Section 2. In Section 3, we describe the density power divergence (DPD) measure and the corresponding estimator for this model. The theoretical properties, including the asymptotic distribution and the influence function of the proposed estimator, are presented in Section 4. We also propose a method to select the optimum DPD parameter by minimizing the asymptotic mean square error of the treatment means. Section 6 illustrates an extensive simulation study based on the proposed method and compares the results with the traditional techniques and other robust methods. The numerical results are further supported through a set of real data examples in Section 7. Some concluding remarks are given in Section 8, and the proofs of the technical results are shown in the Appendix A and Supplementary Materials.

## 2. Generalized Analysis of Variance Model

Let us consider the generalized ANOVA model as follows:

(1) $$y_{ij}=\mu_{i}+\varepsilon_{ij},\;\;i=1,2,\cdots ,k;\;\;j=1,2,\cdots ,n_{i},$$

where $y_{ij}$ is the *j*-th observation under the *i*-th categorical group and $\sum_{i=1}^{k}n_{i}=N$. Here, $\mu_{i}$ is the unobserved fixed effect of the *i*-th group. We assume that the random errors $\varepsilon_{ij}$ are independent random variables with mean zero and finite variance. As we are dealing with a robust estimator, we do not assume that the error term necessarily follows a normal distribution but rather a contaminated normal distribution with *p* proportion outliers, where 0≤p<0.5. However, the target distribution for $\varepsilon_{ij}$ is $N(0,\sigma^{2})$ for all i=1,2,⋯,k and $j=1,2,\cdots ,n_{i}$. Thus, the model parameter $\theta ={\left(\mu_{1},\mu_{2},\cdots ,\mu_{k},\sigma^{2}\right)}^{T}$, with θ∈Θ, is robustly estimated to match the target distribution. We denote the target distribution of $y_{ij}$, i.e., $N(\mu_{i},\sigma^{2})$, as $f_{\theta }\left(y_{ij}|i\right)$, or simply $f_{\theta }\left(y_{ij}\right)$. It is also referred to as the model distribution. The following assumption is needed to define the true data generating distribution.

**Assumption** **1.** *Suppose the true data generating distribution $g(y_{ij})$ contains p proportion outliers from an arbitrary distribution $\chi (y_{ij})$, i.e., $g\left(y_{ij}\right)=\left(1-p\right)f_{\theta_{0}}\left(y_{ij}\right)+p\chi \left(y_{ij}\right)$, where 0≤p<0.5 and $\theta_{0}\in \Theta$. We assume that there exist a small positive number $\gamma_{0}$, such that $\eta \left(\gamma \right)=\max_{i}\int_{y_{ij}}f_{\theta_{0}}^{\gamma }\left(y_{ij}\right)\chi \left(y_{ij}\right)dy_{ij}$ is sufficiently small for $\gamma >\gamma_{0}$.*

A small value of η(γ) ensures that χ(·) is an outlying distribution as the effective mass of χ(·) lies at the tail of the model distribution $f_{\theta }\left(\cdot \right)$ [39]. Here, we relaxed the normality assumption from the classical ANOVA model; however, the main structure of the true distribution should be normal, only the tails may be different. If the main structure of the block distributions is not normal, one may consider a different model for $f_{\theta }\left(y_{ij}\right)$. Although all the calculations in this paper are based on the normal model, one may follow the same techniques for an arbitrary model.

We also eliminate another crucial constraint from the classical ANOVA model: the error distributions are identical. We only need them to be mutually independent. Here, $g(y_{ij})$ is the true density of the *i*-th block, and different blocks may have different variances without violating Assumption 1. Thus, our approach allows heteroscedasticity if the outlying distribution causes it.

## 3. Density Power Divergence

Let us consider a family of models $\left\{F_{\theta },\theta \in \Theta \right\}$ with density $f_{\theta }$. We denote G as the class of all distributions having densities with respect to the Lebesgue measure. Suppose G∈G is the true distribution with density *g*. Then, the DPD measure between the model density $f_{\theta }$ and the true density *g* is defined as follows:

(2) $$d_{\gamma }\left(f_{\theta },g\right)=\left\{\begin{array}{cc} \int_{y}\left\{f_{\theta }^{1+\gamma }\left(y\right)-\left(1+\frac{1}{\gamma }\right)f_{\theta }^{\gamma }\left(y\right)g\left(y\right)+\frac{1}{\gamma }g^{1+\gamma }\left(y\right)\right\}dy, & \mathrm{for}\;\gamma >0,\\ \int_{y}g\left(y\right)\log\left(\frac{g(y)}{f_{\theta }\left(y\right)}\right)dy, & \mathrm{for}\;\gamma =0, \end{array}\right.$$

where γ is a tuning parameter [34]. Note that *G* is not necessarily a member of the model family $F_{\theta }$. Further, for γ=0, the DPD measure is obtained as a limiting case of $\gamma \to 0^{+}$, and is the same as the Kullback-Leibler (KL) divergence. Given a parametric model, we estimate θ by minimizing the DPD measure with respect to θ over its parametric space Θ. We call the estimator the *minimum power divergence estimator* (MDPDE). It is well-known that, for γ=0, minimization of the KL-divergent is equivalent to maximization of the log-likelihood function. Thus, the maximum likelihood estimator (MLE) can be considered a special case of the MDPDE when γ=0.

Let $\theta ={\left(\mu_{1},\mu_{2},\cdots ,\mu_{k},\sigma^{2}\right)}^{T}$ denote the parameter of the generalized ANOVA model (1). We have the model density $f_{\theta }\left(y_{ij}\right)=\frac{1}{\sqrt{2\pi }\sigma }\exp\left\{-\frac{1}{2\sigma^{2}}{\left(y_{ij}-\mu_{i}\right)}^{2}\right\}.$ For γ>0, the DPD measure can empirically be written as

(3) $${\hat{d}}_{\gamma }\left(f_{\theta },g\right)=\frac{1}{N}\sum_{i=1}^{k}\sum_{j=1}^{n_{i}}\int_{y_{ij}}f_{\theta }^{1+\gamma }\left(y_{ij}\right)dy_{ij}-\frac{1+\gamma }{N\gamma }\sum_{i=1}^{k}\sum_{j=1}^{n_{i}}f_{\theta }^{\gamma }\left(y_{ij}\right)+c\left(\gamma \right),$$

where $c\left(\gamma \right)=\frac{1}{N\gamma }\sum_{i=1}^{k}\sum_{j=1}^{n_{i}}\int_{y_{ij}}g^{1+\gamma }\left(y_{ij}\right)dy_{ij}$ does not depend on θ. Using Equation (B.1) in Supplementary Materials, Equation (3) can be written as

(4) $$\begin{array}{cc} {\hat{d}}_{\gamma }\left(f_{\theta },g\right)\; & ={\left(2\pi \right)}^{-\frac{\gamma }{2}}\sigma^{-\gamma }{\left(1+\gamma \right)}^{-\frac{1}{2}}\left[1-\frac{{\left(1+\gamma \right)}^{3/2}}{N\gamma }\sum_{i=1}^{k}\sum_{j=1}^{n_{i}}\exp\left\{-\frac{\gamma }{2\sigma^{2}}{\left(y_{ij}-\mu_{i}\right)}^{2}\right\}\right]+c\left(\gamma \right). \end{array}$$

The MDPDE of θ is then obtained by minimizing ${\hat{d}}_{\gamma }\left(f_{\theta },g\right)$ over θ∈Θ. Note that if the *j*-th observation under the *i*-th block is an outlier, then the value of $f_{\theta }\left(y_{ij}\right)$ is very small compared to other observations. In that case, its contribution in the second term of Equation (3) is negligible when γ>0; thus, the corresponding MDPDE becomes robust against outliers. On the other hand, when γ=0, the KL divergent can be written as ${\hat{d}}_{\gamma }\left(f_{\theta },g\right)=-\sum_{i=1}^{k}\sum_{j=1}^{n_{i}}\log f_{\theta }\left(y_{ij}\right)+d$, where *d* is independent of θ. For an outlying observation, the KL divergence measure diverges as $f_{\theta }\left(y_{ij}\right)\to 0$. Therefore, the MLE breaks down in the presence of outliers as they dominate the loss function. In fact, the tuning parameter γ controls the trade-off between efficiency and robustness of the MDPDE—robustness measure increases if γ increases, but at the same time, efficiency decreases.

The MDPDE of θ is obtained by directly minimizing the DPD measure given in (4). Alternatively, by solving the estimating equations (given in Supplementary Material C), an iterative algorithm for the MDPDE is as follows:

(5) $$\begin{array}{cc} \mu_{i} & =\frac{\sum_{j=1}^{n_{i}}y_{ij}\exp\left\{-\frac{\gamma }{2\sigma^{2}}{\left(y_{ij}-\mu_{i}\right)}^{2}\right\}}{\sum_{j=1}^{n_{i}}\exp\left\{-\frac{\gamma }{2\sigma^{2}}{\left(y_{ij}-\mu_{i}\right)}^{2}\right\}}\;for\;i=1,2,\cdots ,k,\\ \sigma^{2} & =\frac{\sum_{i=1}^{k}\sum_{j=1}^{n_{i}}{\left(y_{ij}-\mu_{i}\right)}^{2}\exp\left\{-\frac{\gamma }{2\sigma^{2}}{\left(y_{ij}-\mu_{i}\right)}^{2}\right\}}{\sum_{i=1}^{k}\sum_{j=1}^{n_{i}}\exp\left\{-\frac{\gamma }{2\sigma^{2}}{\left(y_{ij}-\mu_{i}\right)}^{2}\right\}-\frac{N\gamma }{{\left(1+\gamma \right)}^{3/2}}}. \end{array}$$

The above algorithm needs initial values for $\mu_{i}$ and σ. To protect against outliers, we use the *i*-th block median for $\mu_{i}$ for i=1,2,⋯,k, and a scaled median absolute deviation (MAD) for σ. The following lemma gives the interpretation of the parameter in the contaminated model g(·).

**Lemma** **1.** *Under Assumption 1, if η(γ) is sufficiently small for $\gamma >\gamma_{0}$, then the target parameter that minimizes the DPD measure $d_{\gamma }\left(f_{\theta },g\right)$ is $\theta_{0}$ for all values of $\gamma >\gamma_{0}$.*

If η(γ) is sufficiently small, then, under the contaminated model, $d_{\gamma }\left(f_{\theta_{0}},g\right)$ is the minimum for all θ∈Θ. Thus, the true value of θ is always $\theta_{0}$ for $\gamma >\gamma_{0}$. It ensures that the interpretation of $\theta_{0}$ has the same meaning as the classical ANOVA model where the error distribution is normal. Therefore, we keep the target parameter free of γ in the subsequent sections.

## 4. Asymptotic Distribution of the MDPDE

In this section, we present the asymptotic distribution of the MDPDE when the data generating distribution G(y) is not necessarily a contaminated model. Let us define the score function as $u_{\theta }\left(y_{ij}\right)=\frac{\partial }{\partial \theta }\log f_{\theta }\left(y_{ij}\right)$. For i=1,2,⋯,k and $j=1,2,\cdots ,n_{i}$, we define

(6) $$\begin{array}{cc} \; & J^{\left(ij\right)}=\int_{y_{ij}}u_{\theta }\left(y_{ij}\right)u_{\theta }^{T}\left(y_{ij}\right)f_{\theta }^{1+\gamma }\left(y_{ij}\right)dy_{ij}\\ \; & \;+\int_{y_{ij}}\left\{I_{\theta }\left(y_{ij}\right)-\gamma u_{\theta }\left(y_{ij}\right)u_{\theta }^{T}\left(y_{ij}\right)\right\}\left\{g\left(y_{ij}\right)-f_{\theta }\left(y_{ij}\right)\right\}f_{\theta }^{\gamma }\left(y_{ij}\right)dy_{ij},\\ \; & K^{\left(ij\right)}=\int_{y_{ij}}u_{\theta }\left(y_{ij}\right)u_{\theta }^{T}\left(y_{ij}\right)f_{\theta }^{2\gamma }\left(y_{ij}\right)g\left(y_{ij}\right)dy_{ij}-\xi^{\left(ij\right)}\xi^{(ij)T},\\ \; & I_{\theta }\left(y_{ij}\right)=-\frac{\partial }{\partial \theta }u_{\theta }\left(y_{ij}\right),\;\xi^{\left(ij\right)}=\int_{y_{ij}}u_{\theta }\left(y_{ij}\right)f_{\theta }^{\gamma }\left(y_{ij}\right)g\left(y_{ij}\right)dy_{ij}. \end{array}$$

The form of $u_{\theta }\left(y_{ij}\right)$ is given in Supplementary Material D. We further define

(7) $$J=\lim_{N\to \infty }\frac{1}{N}\sum_{i=1}^{k}\sum_{j=1}^{n_{i}}J^{\left(ij\right)},\;and\;K=\lim_{N\to \infty }\frac{1}{N}\sum_{i=1}^{k}\sum_{j=1}^{n_{i}}K^{\left(ij\right)}.$$

Here, as N→∞, we also need $n_{i}/N\to c_{i}$, such that $c_{i}>0$ for all i=1,2,⋯,k and $\sum_{i}c_{i}=1$. For the consistency and asymptotic distribution of the MDPDE, we need the following assumptions:

(A1) The true density $g(y_{ij})$ is supported over the entire real line R.
(A2) There is an open subset ω∈Θ containing the best fitting parameter θ such that *J* is positive definite for all θ∈ω.
(A3) Suppose $V_{\theta }\left(y_{ij}\right)=\exp\left\{-\frac{\gamma }{2\sigma^{2}}{\left(y_{ij}-\mu_{i}\right)}^{2}\right\}$. There exist functions $M_{rst}\left(y_{ij}\right)$ such that $|\partial^{3}V_{\theta }\left(y_{ij}\right)/\partial \theta_{r}\partial \theta_{s}\partial \theta_{t}|\leq M_{rst}\left(y_{ij}\right)$ for all θ∈ω, where $E_{g}\left(\right|M_{rst}\left(y_{ij}\right)\left|\right)=$ $\int_{y_{ij}}\left|M_{rst}\left(y_{ij}\right)\right|$ $g\left(y_{ij}\right)dy_{ij}<\infty$ for all r,s and *t*.
(A4) We denote δ(·) as the indicator function. Then, for all *r* and *s*, we have
  (8) $$\lim_{\chi \to \infty }\underset{N>1}{\sup}\left\{\frac{1}{N}\sum_{i=1}^{k}\sum_{j=1}^{n_{i}}E_{g}\left[|\frac{\partial }{\partial \theta_{r}}V_{\theta }\left(y_{ij}\right)|\delta \left(|\frac{\partial }{\partial \theta_{r}}V_{\theta }\left(y_{ij}\right)|>\chi \right)\right]\right\}=0,$$
  (9) $$\begin{array}{cc} \lim_{\chi \to \infty }\underset{N>1}{\sup}\{\frac{1}{N} & \sum_{i=1}^{k}\sum_{j=1}^{n_{i}}E_{g}\left[|\frac{\partial^{2}}{\partial \theta_{r}\theta_{s}}V_{\theta }\left(y_{ij}\right)-E_{g}\left(\frac{\partial^{2}}{\partial \theta_{r}\theta_{s}}V_{\theta }\left(y_{ij}\right)\right)|\right.\\ \; & \left.\times \delta \left(|\frac{\partial^{2}}{\partial \theta_{r}\theta_{s}}V_{\theta }\left(y_{ij}\right)-E_{g}\left(\frac{\partial^{2}}{\partial \theta_{r}\theta_{s}}V_{\theta }\left(y_{ij}\right)\right)|>\chi \right)\right]\}=0. \end{array}$$
(A5) Let $K_{N}=\frac{1}{N}\sum_{i=1}^{k}\sum_{j=1}^{n_{i}}K^{\left(ij\right)}$. For all ϵ>0, we have
  (10) $$\lim_{N\to \infty }\left\{\frac{1}{N}\sum_{i=1}^{k}\sum_{j=1}^{n_{i}}E_{g}\left[{∥K_{N}^{-1/2}\frac{\partial }{\partial \theta }V_{\theta }\left(y_{ij}\right)∥}^{2}\right.\delta \left(∥K_{N}^{-1/2}\frac{\partial }{\partial \theta }V_{\theta }\left(y_{ij}\right)∥>\epsilon \sqrt{N}\right)\right]\}=0.$$

Under the independent heterogeneous setup, the above conditions are required to stabilize the matrices *J* and *K* for the existence of the asymptotic distribution (cf. [40,41,42]). These assumptions are satisfied by the true density $g(y_{ij})$ defined in Assumption 1. However, the following theorem is proved for a more general form of $g(y_{ij})$.

**Theorem** **1.** *Under the regularity conditions (A1)–(A5), with probability tending to 1 as N→∞, there exists $\hat{\theta }$, such that*

*(i)*  *$\hat{\theta }$ is consistent for θ, and*
*(ii)*  *the asymptotic distribution of $\hat{\theta }$ is given by*
(11) $$\sqrt{N}\left(\hat{\theta }-\theta \right)∼N_{k+1}\left(0,J^{-1}KJ^{-1}\right).$$

**Proof.**  The proof of the theorem is given in Appendix A.    □

The independent and non-identically distributed samples leading to heterogeneity in variances technically impose a computational burden (cf. [42]). Hence, a positive definite matrix *J* in assumption (A2) is required to stabilize the asymptotic variance of the MDPDE. Furthermore, the assumption (A4) and a generalized version of Khinchin’s weak law of large numbers (cf. [43]) are needed to ensure consistency, while the asymptotic normality is guaranteed by the assumption (A5) and a multivariate extension of the Lindeberg-Levy central limit theorem.

Further calculations in the supplementary materials show that for the uncontaminated model, i.e., when $g=f_{\theta }$, the covariance matrix of $\sqrt{N}\hat{\mu }$ is $\Sigma_{\mu }=\frac{{\left(1+\gamma \right)}^{3}\sigma^{2}}{{\left(1+2\gamma \right)}^{\frac{3}{2}}}\lim_{N\to \infty }S^{-1}$, where *S* is a k×k dimensional diagonal matrix with *i*-th diagonal element $n_{i}/N$. Thus, the variance of each component of $\hat{\mu }$ increases as γ increases. Therefore, the efficiency of the MDPDE decreases as γ increases—the MLE being the most efficient estimator in pure data. However, our simulation studies show that the loss of efficiency is minimal unless γ is too large. On the other hand, the gain in robustness is significant for contaminated data.

### 4.1. Influence Function of the MDPDE

We access the extent of the resistance to outliers of our proposed estimator using the influence function approach of [26]. It measures the rate of asymptotic bias of an estimator to infinitesimal contamination in the distribution. A bounded influence function suggests that the corresponding estimator is robust against extreme outliers. Note that the MDPDE is an M-estimator [25] as the estimating equation can be written as $\sum_{i}\sum_{j}\Psi_{\theta }\left(y_{ij}\right)=0$, where

(12) $$\Psi_{\theta }\left(y_{ij}\right)=u_{\theta }\left(y_{ij}\right)f_{\theta }^{\gamma }\left(y_{ij}\right)-\int_{y}u_{\theta }\left(y_{ij}\right)f_{\theta }^{1+\gamma }\left(y_{ij}\right)dy_{ij}.$$

This is obtained by differentiating ${\hat{d}}_{\gamma }\left(f_{\theta },g\right)$ with respect to θ in Equation (3). Let G(y) be the true distribution function *Y*, and $\theta =T_{\gamma }\left(G\right)$ be functional for the MDPDE. Following [34], the influence function of the MDPDE is given by

(13) $$IF\left(y_{ij},T_{\gamma },G\right)=J^{-1}\left\{u_{\theta }\left(y_{ij}\right)f_{\theta }^{\gamma }\left(y_{ij}\right)-\xi^{\left(ij\right)}\right\},$$

where *J* is evaluated at the model when $g=f_{\theta }$, and $\xi^{\left(ij\right)}$, given in Equation (E.6), is a fixed vector that does not depend on index *i* and *j*.

**Remark** **1.** *Note that the score function $u_{\theta }\left(y_{ij}\right)$ in Equation (D.3) of the Supplementary Materials is unbounded in $y_{ij}$. As a result, the influence function of the MLE, i.e., the MDPDE with γ=0, is unbounded. On the other hand, $u_{\theta }\left(y_{ij}\right)f_{\theta }^{\gamma }\left(y_{ij}\right)$ is bounded in $y_{ij}$ when γ>0 as the corresponding terms can be written as $y_{ij}\exp\left(y_{ij}^{2}\right)$. So, the influence function of the MDPDE of θ is bounded in $y_{ij}$ when γ>0. Moreover, $IF(y_{ij},T_{\gamma },G)$ tends to zero as $|y_{ij}|\to \infty$, indicating a redescending effect for large vertical outliers. The higher the value of γ, the larger the down-weighting effect on the outliers.*

### 4.2. Choice of the Optimum γ

One important use of the asymptotic distribution of the MDPDE is the selection of the optimum value of the DPD parameter γ. As the performance of the test depends on the corresponding estimator, we choose γ that is optimum in terms of robustness and efficiency of $\hat{\mu }={\left({\hat{\mu }}_{1},{\hat{\mu }}_{2},\cdots ,{\hat{\mu }}_{k}\right)}^{T}$. In practice, the user may work with a fixed value of γ depending on the desired level of robustness measure at the cost of efficiency. Alternatively, we may select a data-driven optimum γ. Following [44], we minimize the mean square error (MSE) of $\hat{\mu }$ to obtain the optimum value of γ adaptively. Suppose $\Sigma_{\mu }$ is the asymptotic variance of $\hat{\mu }$ obtained from Theorem 1, assuming that the true distribution belongs to the model family. Let ${\hat{\Sigma }}_{\mu }$ be the estimate of $\Sigma_{\mu }$. The empirical estimate of the MSE, as the function of a pilot estimator $\mu^{P}$, is given by

(14) $$\hat{MSE}\left(\gamma \right)={\left(\hat{\mu }-\mu^{P}\right)}^{T}\left(\hat{\mu }-\mu^{P}\right)+\mathrm{tr}\left({\hat{\Sigma }}_{\mu }\right).$$

From Supplementary Material H, we find that ${\hat{\Sigma }}_{\mu }=\frac{{\left(1+\gamma \right)}^{3}{\hat{\sigma }}^{2}}{{\left(1+2\gamma \right)}^{\frac{3}{2}}}S^{-1}$. In particular, we recommend that a robust estimator, such as the MDPDE with γ∈(0.3,0.5), can be used as a pilot estimator. One should then iterate this process by taking the previous stage’s optimum γ as the current stage’s pilot estimator and proceeding until convergence. It eliminates the sensitivity in the initial value of $\mu^{P}$ as long as the initial estimate is robust. In our numerical examples, we have used this iterative procedure.

Lemma 1 shows that the target parameter is the same for all γ for the contaminated model. Moreover, Theorem 1 proves that all $\hat{\mu }$ converge to the target parameter. However, their small sample performance may be different depending on the contaminated proportion (*p*) and closeness of the contaminated distribution (χ) to the model distribution ($f_{\theta }$). Thus, selecting the DPD parameter γ in finite samples is important to get the best performance.

## 5. Testing of Hypothesis

Let us now consider the ANOVA test, where the null hypothesis assumes no treatment effects, i.e.,

(15) $$H_{0}:\mu_{1}=\mu_{2}=\cdots =\mu_{k}\;\text{against}\;H_{1}:H_{0}\;\text{is not true}.$$

The following m(·) function imposes k−1 restrictions for the null hypothesis:

(16) $$m\left(\theta \right)={\left(\mu_{1}-\mu_{2},\mu_{2}-\mu_{3},\cdots ,\mu_{k-1}-\mu_{k}\right)}^{T}=0_{k-1},$$

where $0_{k-1}$ is a zero vector of length k−1.

**Definition** **1.** *Let $\hat{\theta }$ be the MDPDE of θ. The family of proposed Wald-type test statistics for testing the null hypothesis in (15) is given by*

(17) $$W_{N}=Nm^{T}\left(\hat{\theta }\right){\left[M^{T}\left(\hat{\theta }\right)J^{-1}\left(\hat{\theta }\right)K\left(\hat{\theta }\right)J^{-1}\left(\hat{\theta }\right)M\left(\hat{\theta }\right)\right]}^{-1}m\left(\hat{\theta }\right),$$

*where $M\left(\theta \right)=\frac{\partial m^{T}(\theta )}{\partial \theta }$.*

When γ=0, the Wald-type test statistic reduces to the classical Wald test for testing the null hypothesis in (15). We define a k×(k−1)-dimensional matrix

(18) $$M_{\mu }=\left[\begin{array}{cccccccc} 1 & 0 & 0 & 0 & \cdots & 0 & 0 & 0\\ -1 & 1 & 0 & 0 & \cdots & 0 & 0 & 0\\ 0 & -1 & 1 & 0 & \cdots & 0 & 0 & 0\\ \vdots & \vdots & \vdots & \vdots & \cdots & \vdots & \vdots & \vdots \\ 0 & 0 & 0 & 0 & \cdots & 0 & -1 & 1\\ 0 & 0 & 0 & 0 & \cdots & 0 & 0 & -1 \end{array}\right].$$

Then, the (k+1)×(k−1)-dimensional matrix M(θ) is written as $M\left(\theta \right)={\left(M_{\mu }^{T},0_{k-1}\right)}^{T}$. Using Equation (H.8) from the Supplementary Materials, the test statistic $W_{N}$ in Equation (17) is simplified as

(19) $$W_{N}=N{\hat{\sigma }}^{-2}{\left(1+\gamma \right)}^{-3}{\left(1+2\gamma \right)}^{\frac{3}{2}}m^{T}\left(\hat{\theta }\right){\left[M_{\mu }^{T}S^{-1}M_{\mu }\right]}^{-1}m\left(\hat{\theta }\right),$$

where *S* is a k×k dimensional diagonal matrix with *i*-th diagonal element $n_{i}/N$. In the following theorem, we present the asymptotic distribution of $W_{N}.$

**Theorem** **2.** *The asymptotic null distribution of the proposed Wald-type test statistics given in (19) is chi-square with k−1 degrees of freedom.*

**Proof.**  The proof follows from Theorem 1 using the derivation given in [45].    □

## 6. Numerical Results

To investigate the empirical performance of our proposed method, an extensive simulation study under different sample sizes, block sizes, error distributions, and outlier types is performed. The performance of the proposed method is compared with the classical ANOVA test and two robust alternative methods based on Huber’s M-estimator and Tukey’s MM-estimator [25]. The latter two tests are implemented in R by combining the ‘rlm’ and ‘Anova’ functions from the ‘MASS’ and ‘car’ packages, respectively. For those estimators, we have used the default tuning parameters given in the corresponding functions. The robustness properties of the MDPDE depend on the choice of the tuning parameter, and thus, four fixed values of γ=0.1,0.2,0.3, and 0.4 are considered. The optimum value of γ is determined based on the data-driven adaptive choice of γ as discussed in Section 4.2 and the pilot estimator is used iteratively until convergence. From now on, the DPD with optimum γ is abbreviated as “DPD(Opt.)”.

### 6.1. Levels for Different Block Sizes and Error Distributions

We consider the generalized ANOVA model in (1) with k=3 blocks of sizes; $n_{1}=30,n_{2}=25$, and $n_{3}=35$. First, we consider the standard normal errors $\varepsilon_{ij}∼$N(0,1) for all *i* and *j*. To check the empirical levels of different tests, the dataset is generated from the null hypothesis where $\mu_{1}=\mu_{2}=\mu_{3}=0$. The empirical level is computed as the proportion of test statistics in 5000 replications that exceed the nominal $\chi^{2}$ critical value at a 5% level of significance. The results are reported in the first column of Table 1. From the results, all the values are close to the nominal level. In addition, the MSE of $\hat{\mu }$ (times *N*) for all the estimators is reported in the second column of Table 1. The ANOVA test is based on the MLE, theoretically the most efficient estimator under normal errors. The simulated results also show that the MLE gives the smallest MSE in pure data. The MSE of the MDPDE increases as the value of γ increases. In DPD(Opt.), we minimize the MSE of the block means ($\hat{\mu }$), and the mean value of optimum γ comes out to be 0.0507, which is close to zero. However, as the algorithm uses a dummy value of the true parameter $\mu^{P}$ iteratively, its efficiency is lower than the actual fixed γ that produces the minimum MSE. Thus, the corresponding empirical level is also slightly inflated.

In the following simulation, the error distribution is changed to the Cauchy distribution, and we consider an additional block of size $n_{4}=20$. The empirical levels and MSEs computed for this case are reported in the third and fourth columns of Table 1, respectively. From the results, the MLE breaks down, and the *N* times MSE of $\hat{\mu }$ becomes $1.3\times 10^{10}$ when the errors are heavy-tailed. The MDPDEs with small γ are affected by the heavy-tailed errors, and the corresponding tests become very conservative. On the other hand, the DPD tests with higher values of γ properly maintain the level of the test. The mean value of optimum γ is 0.5865. So, the algorithm adaptively selects a higher value of γ as the data contains some extreme values. The tests based on Huber and Tukey’s estimators properly maintain the level of the test; however, the MSEs of $\hat{\mu }$ are much higher than DPD(Opt.).

Furthermore, we consider two additional cases with k=5 and k=6, where additional block sizes are $n_{5}=30$ and $n_{6}=50$. The error distributions are the standard normal and *t*-distribution with 3 degrees of freedom ($t_{3}$), respectively. The results from the third simulation with k=5 are similar to the first case with k=3. On the other hand, the MSE of the MLE is still too large in the fourth case as $t_{3}$ is a heavy-tailed distribution, although less extreme than the Cauchy distribution. The empirical level of the ANOVA test improves; however, as demonstrated in the later part of our numerical results, the power of the test is considerably affected in such situations.

**Remark** **2.** *Note that in our numerical analyses, the Cauchy and t-distributions, which follow the form of the true density $g(y_{ij})$ in Assumption 1, are considered as the structure of the central region resembles the normal model, i.e., only the tails are different. On the other hand, a chi-square error distribution with smaller degrees of freedom deviates much from the normal model, and thus, it creates a discrepancy in the empirical levels and loss of power for the DPD tests. As discussed in Section 2, in such cases, one needs to assume a different model for $f_{\theta }\left(y_{ij}\right)$ and compute the test statistic accordingly.*

### 6.2. Levels for Different Sample Sizes

**Figure 1:**
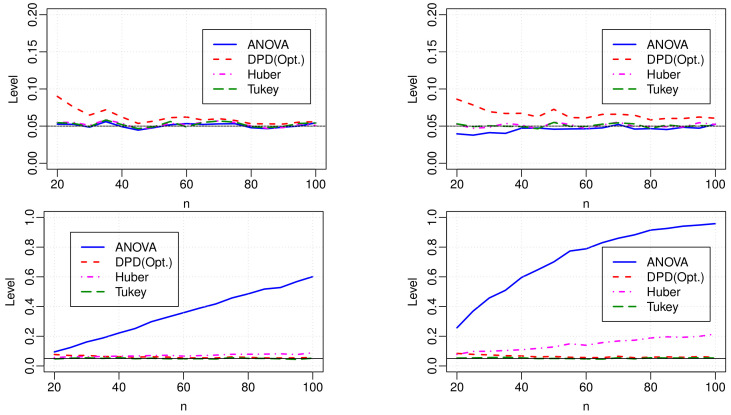
The level of different tests in pure data (**top left**) and in the presence of 5% vertical outliers at random locations (**top right**), 5% clustered outliers (**bottom left**), and 10% clustered outliers (**bottom right**). In all cases, k=4.

Let us consider the generalized ANOVA Model in (1) with k=4 blocks and equal sample size (*n*) in all blocks, i.e., $n_{1}=n_{2}=n_{3}=n_{4}=n$. The performance of the estimators is examined under the standard normal errors $\varepsilon_{ij}∼$ N(0,1) for the increasing number of sample size per block (n=20 to 100). The plot at the top left in Figure 1 displays the empirical levels of all tests for different values of the sample sizes. To avoid overlapping plots, we present the results only for one DPD test, DPD(Opt.), excluding the tests with fixed γ. From the results, DPD(Opt.) shows inflated levels, but it settles down rapidly around the nominal level as the sample size increases. Other tests, including the DPDs with fixed γ (not presented in the plot), perform well in maintaining the level of the test even in small sample sizes.

### 6.3. Effect of Outliers

In the following setups, the robustness properties of the estimators are evaluated in the presence of different types of outliers. A certain percentage (*p*%) of outliers are inserted using two different scenarios to generate contaminated data; (a) random contamination and (b) concentrated contamination. Following Assumption 1, the contamination schemes are as follows.

1. The random outliers in the *y*-direction, i.e., random vertical outliers, are obtained by replacing *p*% original standard normal errors with $\varepsilon_{it}∼$ N(10,1) in the generalized ANOVA model (1).
2. Concentrated vertical outliers are generated by substituting *p*% errors in the first block by $\varepsilon_{it}∼$ N(10,1).

The plots at the top right, bottom left, and bottom right in Figure 1 present the empirical levels of different tests in contaminated data. The plot on the top right in Figure 1 presents the results when the dataset is contaminated at random locations with 5% outliers. In this case, all the methods produce similar performance with their performance obtained when no outlier is present in the data (i.e., the plot on the top left in Figure 1). Outliers do not alter the level even for the classical ANOVA test, as all the blocks are equally affected by the outliers. DPD(Opt.) is slightly liberal as the optimum γ is estimated from the data. In the bottom plots, the first block is contaminated by the clustered outliers with 5% (left) and 10% (right) contamination levels. The results indicate that the clustered outliers drastically inflate the empirical levels of the ANOVA test. The Huber test eventually fails to maintain its level when the proportion of clustered outliers is large. On the other, the empirical levels of the DPD(Opt.) test are very close to the nominal level.

### 6.4. Empirical Powers

**Figure 2:**
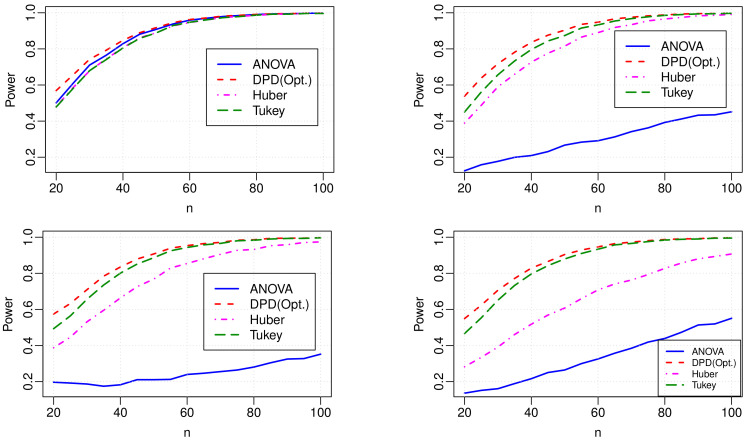
The power of different tests in pure data (**top left**) and in the presence of 5% vertical outliers at random locations (**top right**), 5% clustered outliers (**bottom left**) and 10% clustered outliers (**bottom right**). In all cases, k=4 and $\mu ={\left(-0.4,0.2,-0.1,0.3\right)}^{T}$.

The empirical powers of the test procedures for all the outlier types and contamination levels are presented in Figure 2. To compute the power of the tests, the dataset is generated under the alternative hypothesis using the block means $\mu ={\left(-0.4,0.2,-0.1,0.3\right)}^{T}$. The top left plot in Figure 2 shows that the power of all four tests is similar when no outlier is present in the data, and the power converges to one as the sample size increases. DPD(Opt.) shows slightly higher power in small sample sizes. However, the level corrected power (not presented in the plot) is equivalent to other tests. From other plots in Figure 2, the classical ANOVA test is severely affected by both types of outliers. While the power of the Huber test is relatively high, it loses sufficient power, especially in the presence of clustered outliers at a large percentage. In other words, the Huber test is not fully robust to the clustered outliers. Compared with other tests, the proposed DPD(Opt.) produces improved power values in all cases. Moreover, it produces higher power that is not affected by the outlier types and contamination levels. While Tukey’s test produces higher power than the classical ANOVA, the proposed DPD test gives even better power than Tukey’s test even after level correction.

In a nutshell, the results produced by our simulation studies suggest that the performance of the proposed DPD test is similar to the classical ANOVA test when no outlier is present in the data. On the other hand, the DPD test with large values of γ yields an improved level and power values than the classical ANOVA and the test based on the Huber estimator. In addition, the data-dependent optimum MDPDE successfully produces the optimum performance and adequately balances the efficiency in pure data and robustness properties in the contaminated data. Moreover, our results indicate that the proposed method produces a competitive or even better level and power than the tests based on other M-estimators.

**Remark** **3.** *We note that the error distributions are not identically distributed in the case of the clustered outliers. In addition, the error variance is different in the first block, i.e., the model is heteroscedastic because of the outliers. In this case, some of the assumptions for the classical ANOVA test are not satisfied, and thus, the empirical level breaks down, and the test losses significant power. On the other hand, our proposed DPD test produces consistent results and successfully relaxes those assumptions.*

## 7. Case Study

### 7.1. Bone Marrow Transplant Dataset

**Figure 3:**
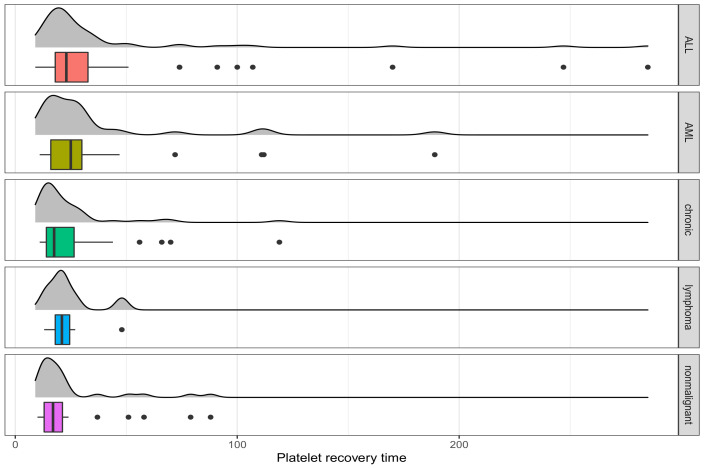
The box-plots and normal kernel density plots of platelet recovery time (in days) for different group of patients in the bone marrow transplant dataset.

The bone marrow transplant dataset, originally reported by [46], describes several hematologic diseases for 187 children and adolescents (112 males and 75 females) diagnosed with malignant (n=155) and nonmalignant disorders (n=32). The patients underwent unmanipulated allogeneic unrelated donor hematopoietic stem cell transplantation between 2000 and 2008. Their median age at transplant is 9.6 years (range: 0.6–20.2 years). With this dataset, our aim is to test if the average time to platelet recovery is related to the type of hematologic diseases. The platelet recovery is defined as a recovery of platelet count greater than $50\times 10^{9}$/L without transfusion support for three consecutive days. The first day of three consecutive days is regarded as the day of platelet engraftment. Because of some missing cases or patients without platelet recovery, 17 observations containing platelet recovery time of $10^{6}$ days are excluded from the data. The remaining dataset has 142 patients with malignant disorders and 28 nonmalignant cases. Among the malignant disorders patients, there are cases of 62 acute lymphoblastic leukemia (ALL), 31 acute myelogenous leukemia (AML), 42 chronic myelogenous leukemia, and 7 lymphomas. Figure 3 presents the box plots and normal kernel density plots of platelet recovery time (in days) for different groups of patients. From Figure 3, the dataset has some large outliers, which motivates us to apply our proposed method to robustly test the equality of the average times to platelet recovery of different types of hematologic diseases.

The results are presented in Table 2. In this table, the first four columns denote the results of the ANOVA, DPD(Opt.), Huber, and Tukey’s tests, respectively. From the results, the ANOVA test based on the MLE is considerably affected by the outliers, and the computed block means for this method are higher than the other methods. Note that the block medians of the five groups are 23, 25, 17.5, 21, and 17, respectively. In addition, the MLE produces a considerably larger standard deviation, $\hat{\sigma }=36.83$, compared with the robust methods, yielding a large *p*-value = 0.2298 for the ANOVA test. On the other hand, the robust methods produce smaller estimates for σ. Thus, the *p*-values obtained by the DPD and Tukey’s tests are significant at a 5% level of significance, while Huber’s test is on the borderline.

In this dataset, the block lymphoma has only seven patients, i.e., it includes only 4.12% of observations. Therefore, the test results may be biased due to the unbalanced case. To overcome this problem, we remove this block and re-compute the results for all the methods. The results are presented in the last four columns of Table 2. From the results, the *p*-value computed from the ANOVA is still large. On the other hand, the robust tests comfortably reject the null hypothesis at a 5% level of significance. Consequently, the results indicate that the platelet recovery time varies significantly depending on the type of hematologic disease. However, the classical ANOVA test fails to detect the difference because of the impacts of the large outliers.

### 7.2. Glucose Level Dataset

**Figure 4:**
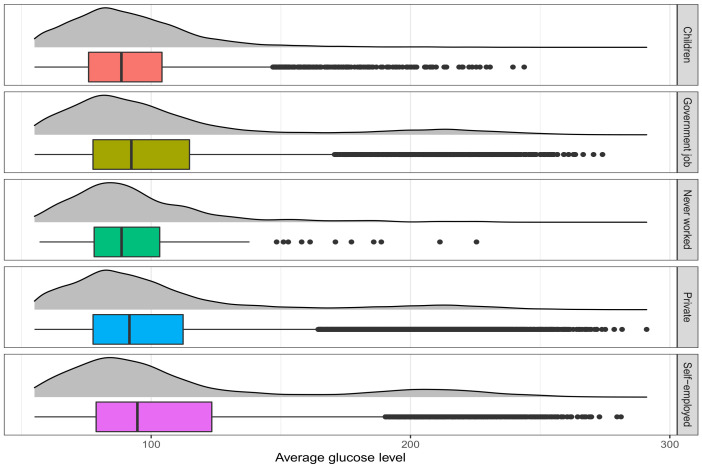
The box-plots and normal kernel density plots of average glucose level for different groups in the glucose level dataset.

We analyze the glucose level dataset, where we are interested in determining the significant difference in average blood glucose levels among work types. The original dataset, available at https://healthdata.gov/ (accessed on 28 July 2022), is used to predict cerebral stroke based on 11 features (see [47]). The dataset contains 43,400 observations, including 6156 children aged below 16. The adults are categorized into four groups based on their work type—government, never worked, private, and self-employed. Figure 4 presents the box plots and normal kernel density plots of average glucose levels for different groups. This figure denotes that all the distributions have long tails to the right. In other words, the population of each group may follow a contaminated normal distribution. Therefore, it is expected that the robust tests may produce better results compared to the classical ANOVA.

Our results for the glucose level dataset are presented in the first four columns of Table 3. The null hypothesis claims that the means of the average glucose level in all categories are equal. All the tests excluding the proposed DPD(Opt.) come out to be significant as the *p*-values are almost identical to zero. Here, the group medians are 88.52, 92.35, 88.57, 91.61, and 94.68, respectively. From Table 3, the group means computed by the ANOVA are inflated dramatically because of the outliers. On the other hand, the estimates of the group mean obtained by the DPD(Opt.) are not significantly different.

Similar to the bone marrow transplant dataset, this dataset is also unbalanced as only 117 people (0.41% of the sample size) have never worked. The other categories have sufficient sample sizes—6156 children, 5440 government jobs, 24,834 private jobs, and 6793 self-employed. Thus, the 117 observations belonging to the category ‘never worked’ are discarded from the dataset to obtain a balanced design. The results obtained for this balanced case are presented in the last four columns of Table 3. From the results, the computed *p*-values obtained by ANOVA ($1.2\times 10^{-161}$) and Huber ($3.7\times 10^{-61}$) tests are still very small. In this case, Tukey’s test produces a large *p*-value (0.2890), and the corresponding estimates of the group means are close to 90. The *p*-value obtained by the DPD test is reduced compared with the one obtained from the unbalanced case, but it is still insignificant at the 5% level. Thus, it is evident from the results that the ANOVA and Huber tests produce false positive results for this dataset. On the other hand, the proposed and Tukey’s tests show strong robustness against outliers.

## 8. Conclusions

In this study, we propose a robust procedure for testing the main effect in the one-way ANOVA model under mild restrictions. The test has a tuning parameter that controls the efficiency and robustness of the MDPDE of the treatment effect. In addition, we propose an adaptive method that estimates the tuning parameter without prior knowledge of the outliers. The proposed test can be used even if the normality assumption is violated at the tails of the distribution or errors are heteroscedastic because of the outliers. The empirical performance of the proposed method is evaluated via an extensive simulation study, and the results are favorably compared with existing robust and non-robust testing procedures. Our results indicate that the proposed method produces similar results to the classical ANOVA when no outlier is present in the data. On the other hand, the proposed method produces competitive or even significantly better results than the existing robust methods when outliers contaminate the data. Through several empirical data examples, we demonstrate that the proposed test can uncover both masking effects caused by outliers—blurring the actual difference when one exists and detecting a difference when none exists.

There are several ways in which the present study can be further extended. For instance, using a flexible formulation of the hypotheses obtained by a convenient contrast matrix as discussed by [23]; the proposed test can be extended to the more complex structure of the designs, such as factorial ANOVA. In addition, the proposed method can be used with the non-parametric inference procedures, such as the one proposed by [48], to incorporate the uncertainty associated with the underlying effect estimators and to handle the right-censored survival data.

## Figures and Tables

**Table 1:** The empirical level of different tests and the MSE of $\hat{\mu }$ (times *N*) for the corresponding estimators for different block sizes and error distributions.

Tests	k=3, Normal		k=4, Cauchy		k=5, Normal		k=6, $t_{3}$	
	Level	MSE	Level	MSE	Level	MSE	Level	MSE
ANOVA	0.0480	8.9155	0.0218	$1.3\times 10^{10}$	0.0464	26.0063	0.0410	114.8192
DPD (0.1)	0.0592	9.0236	0.0310	103.4623	0.0594	26.2686	0.0394	69.7182
DPD (0.2)	0.0574	9.3041	0.0402	61.7873	0.0578	27.0460	0.0432	62.8766
DPD (0.3)	0.0580	9.7067	0.0462	49.7004	0.0594	28.1916	0.0506	60.7463
DPD (0.4)	0.0606	10.2002	0.0498	44.5338	0.0650	29.6157	0.0570	60.5194
DPD (Opt.)	0.0652	9.2297	0.0602	41.4773	0.0638	26.4132	0.0654	61.9717
Huber	0.0480	9.4081	0.0468	73.6107	0.0486	27.3243	0.0468	62.5188
Tukey	0.0480	9.4475	0.0462	52.4656	0.0480	27.3652	0.0484	62.0656

**Table 2:** The parameter estimates for the generalized ANOVA model and the *p*-values of different tests for the bone marrow transplant dataset. The last four columns give results when lymphoma patients are removed from the dataset.

	Full Data				Reduced Data			
	ANOVA	DPD (Opt.)	Huber	Tukey	ANOVA	DPD(Opt.)	Huber	Tukey
ALL (${\hat{\mu }}_{1}$)	38.85	21.28	24.84	22.00	38.85	21.36	24.85	22.02
AML (${\hat{\mu }}_{2}$)	35.52	21.33	24.39	21.93	35.52	21.38	24.41	21.94
Chronic (${\hat{\mu }}_{3}$)	24.55	17.71	20.11	18.27	24.55	17.77	20.12	18.28
Lymphoma (${\hat{\mu }}_{4}$)	23.86	20.12	22.11	20.99	–	–	–	–
Non-malignant (${\hat{\mu }}_{5}$)	24.39	16.24	19.05	16.58	24.39	16.27	19.07	16.59
$\hat{\sigma }$	36.83	7.01	10.14	8.04	37.45	7.12	10.20	8.09
*p*-value	0.2298	0.0101	0.0511	0.0132	0.1644	0.0045	0.0263	0.0058

**Table 3:** The parameter estimates for the generalized ANOVA model and the *p*-values of different tests for the glucose level dataset. The last four columns give results when the ‘Never worked’ category is removed from the dataset.

	Full Data				Reduced Data			
	ANOVA	DPD(Opt.)	Huber	Tukey	ANOVA	DPD(Opt.)	Huber	Tukey
Children (${\hat{\mu }}_{1}$)	92.38	89.02	90.41	90.40	92.38	89.40	90.41	89.82
Government job (${\hat{\mu }}_{2}$)	107.10	89.21	95.85	91.89	107.10	89.94	95.86	90.15
Never worked (${\hat{\mu }}_{3}$)	94.70	89.04	91.39	90.97	–	–	–	–
Private (${\hat{\mu }}_{4}$)	104.78	89.26	94.95	91.76	104.78	89.94	94.95	90.20
Self-employed (${\hat{\mu }}_{5}$)	112.51	89.66	98.60	93.12	112.51	90.56	98.61	90.61
$\hat{\sigma }$	42.74	22.07	25.76	28.39	42.79	23.06	25.78	23.59
*p*-value	8.8 $\times 10^{-163}$	0.6231	7.5 $\times \;10^{-61}$	1.5 $\times \;10^{-7}$	1.2 $\times \;10^{-161}$	0.0537	3.7 $\times \;10^{-61}$	0.2890

## Data Availability

The bone marrow transplant dataset can be found here: https://archive.ics.uci.edu/ml/datasets/Bone+marrow+transplant%3A+children (accessed on 28 July 2022), and the glucose level dataset can be found here: https://www.kaggle.com/datasets/shashwatwork/cerebral-stroke-predictionimbalaced-dataset (accessed on 28 July 2022).

## Supplementary Materials

The following supporting information can be downloaded at: https://www.mdpi.com/article/10.3390/e24091189/s1.

## Appendix A. Proof of Theorem 1

The proof of the first part closely follows the consistency of the maximum likelihood estimator with the line of modifications as given in Theorem 3.1 of [40]. For brevity, we only present the detailed proof of the second part.

Let $\hat{\theta }$ be the MDPDE of θ. Then

(A1) $$\frac{\partial }{\partial \theta }{\hat{d}}_{\gamma }\left(f_{\theta },g\right)=0.$$

Differentiating Equation (3), it can be written as an M-estimator as follows

(A2) $$\sum_{i=1}^{k}\sum_{j=1}^{n_{i}}\Psi_{\hat{\theta }}\left(y_{ij}\right)=0,$$

where

(A3) $$\Psi_{\theta }\left(y_{ij}\right)=u_{\theta }\left(y_{ij}\right)f_{\theta }^{\gamma }\left(y_{ij}\right)-\int_{y}u_{\theta }\left(y\right)f_{\theta }^{1+\gamma }\left(y_{ij}\right)dy.$$

Let $\theta_{g}$ be the true value of θ, then

(A4) $$\begin{array}{cc} E\left(\sum_{i=1}^{k}\sum_{j=1}^{n_{i}}\Psi_{\theta_{g}}\left(y_{ij}\right)\right) & =0\\ \Rightarrow \sum_{i=1}^{k}\sum_{j=1}^{n_{i}}[\int_{y}u_{\theta_{g}}\left(y\right)f_{\theta_{g}}^{\gamma }\left(y\right)g\left(y\right)dy & -\int_{y}u_{\theta_{g}}\left(y\right)f_{\theta_{g}}^{1+\gamma }\left(y\right)dy]=0 \end{array}$$

Taking a Taylor series expansion of Equation (A2), we get

(A5) $$\begin{array}{cc} \frac{1}{N}\sum_{i=1}^{k}\sum_{j=1}^{n_{i}}\Psi_{\theta_{g}}\left(y_{ij}\right) & +\frac{1}{N}\sum_{i=1}^{k}\sum_{j=1}^{n_{i}}\frac{\partial }{\partial \theta }\Psi_{\theta }\left(y_{ij}\right){|}_{\theta =\theta_{g}}\left(\hat{\theta }-\theta_{g}\right)+R_{N}=0\\ \sqrt{N}\left(\hat{\theta }-\theta_{g}\right) & =-{\left[\frac{1}{N}\sum_{i=1}^{k}\sum_{j=1}^{n_{i}}\frac{\partial }{\partial \theta }\Psi_{\theta }\left(y_{ij}\right){|}_{\theta =\theta_{g}}\right]}^{-1}\left[\frac{1}{\sqrt{N}}\sum_{i=1}^{k}\sum_{j=1}^{n_{i}}\Psi_{\theta_{g}}\left(y_{ij}\right)+\sqrt{N}R_{N}\right]. \end{array}$$

Under regularity condition (A1)–(A3), it can be easily shown that the reminder term $\sqrt{N}R_{N}=o_{p}\left(1\right).$ Now, we will show that

(A6) $$\begin{array}{c} \frac{1}{N}\sum_{i=1}^{k}\sum_{j=1}^{n_{i}}\frac{\partial }{\partial \theta }\Psi_{\theta }\left(y_{ij}\right){|}_{\theta =\theta_{g}}\overset{p}{\to }-J,\;and\;\frac{1}{\sqrt{N}}\sum_{i=1}^{k}\sum_{j=1}^{n_{i}}\Psi_{\theta_{g}}\left(y_{ij}\right)\overset{a}{∼}N\left(0,K\right). \end{array}$$

Therefore, from Equation (A5), we will prove the theorem as

(A7) $$\sqrt{N}\left(\hat{\theta }-\theta_{g}\right)\overset{a}{∼}N\left(0,J^{-1}KJ^{-1}\right).$$

First Part:

(A8) $$\begin{array}{cc} \; & \frac{1}{N}\sum_{i=1}^{k}\sum_{j=1}^{n_{i}}\frac{\partial }{\partial \theta }\Psi_{\theta }\left(y_{ij}\right)\\ \; & \overset{p}{\to }\lim_{N\to \infty }E\left[\frac{1}{N}\sum_{i=1}^{k}\sum_{j=1}^{n_{i}}\frac{\partial }{\partial \theta }\Psi_{\theta }\left(y_{ij}\right)\right]\\ \; & \overset{p}{\to }\lim_{N\to \infty }\frac{1}{N}\sum_{i=1}^{k}\sum_{j=1}^{n_{i}}E\left[\frac{\partial }{\partial \theta }\left(u_{\theta }f_{\theta }^{\gamma }-\int u_{\theta }f_{\theta }^{1+\gamma }\right)\right]\\ \; & \overset{p}{\to }\lim_{N\to \infty }\frac{1}{N}\sum_{i=1}^{k}\sum_{j=1}^{n_{i}}E\left[-I_{\theta }f_{\theta }^{\gamma }+\gamma u_{\theta }u_{\theta }^{T}f_{\theta }^{\gamma }-\int \left\{-I_{\theta }f_{\theta }^{1+\gamma }+\left(1+\gamma \right)u_{\theta }u_{\theta }^{T}f_{\theta }^{1+\gamma }\right\}\right]\\ \; & \overset{p}{\to }\lim_{N\to \infty }\frac{1}{N}\sum_{i=1}^{k}\sum_{j=1}^{n_{i}}\left[-\int I_{\theta }f_{\theta }^{\gamma }g+\gamma \int u_{\theta }u_{\theta }^{T}f_{\theta }^{\gamma }g+\int I_{\theta }f_{\theta }^{1+\gamma }-\left(1+\gamma \right)\int u_{\theta }u_{\theta }^{T}f_{\theta }^{1+\gamma }\right]\\ \; & \overset{p}{\to }-\lim_{N\to \infty }\frac{1}{N}\sum_{i=1}^{k}\sum_{j=1}^{n_{i}}\left[\int u_{\theta }u_{\theta }^{T}f_{\theta }^{1+\gamma }+\int \left(I_{\theta }-\gamma u_{\theta }u_{\theta }^{T}\right)\left(g-f_{\theta }\right)f_{\theta }^{\gamma }\right]. \end{array}$$

So Equation (6) gives

(A9) $$\frac{1}{N}\sum_{i=1}^{k}\sum_{j=1}^{n_{i}}\frac{\partial }{\partial \theta }\Psi_{\theta }\left(y_{ij}\right){|}_{\theta =\theta_{g}}\overset{p}{\to }-\lim_{N\to \infty }\frac{1}{N}\sum_{i=1}^{k}\sum_{j=1}^{n_{i}}J^{\left(ij\right)}=-J.$$

Second Part: From Equation (A4), we get

(A10) $$\begin{array}{cc} E\left[\frac{1}{\sqrt{N}}\sum_{i=1}^{k}\sum_{j=1}^{n_{i}}\Psi_{\theta_{g}}\left(y_{ij}\right)\right]=\frac{1}{\sqrt{N}}\sum_{i=1}^{k}\sum_{j=1}^{n_{i}}[\int_{y_{ij}} & u_{\theta_{g}}\left(y_{ij}\right)f_{\theta_{g}}^{\gamma }\left(y_{ij}\right)g\left(y_{ij}\right)dy_{ij}\\ \; & -\int_{y}u_{\theta_{g}}\left(y_{ij}\right)f_{\theta_{g}}^{1+\gamma }\left(y_{ij}\right)dy_{ij}]=0. \end{array}$$

Now

(A11) $$\begin{array}{cc} V\left[\frac{1}{\sqrt{N}}\sum_{i=1}^{k}\sum_{j=1}^{n_{i}}\Psi_{\theta_{g}}\left(y_{ij}\right)\right]\; & =\frac{1}{N}\sum_{i=1}^{k}\sum_{j=1}^{n_{i}}V\left[\Psi_{\theta_{g}}\left(y_{ij}\right)\right]\\ \; & =\frac{1}{N}\sum_{i=1}^{k}\sum_{j=1}^{n_{i}}\left[\int_{y}u_{\theta_{g}}\left(y_{ij}\right)u_{\theta_{g}}^{T}\left(y_{ij}\right)f_{\theta_{g}}^{2\gamma }\left(y_{ij}\right)g\left(y_{ij}\right)dy_{ij}-\xi^{\left(i\right)}\xi^{(i)T}\right]\\ \; & =\frac{1}{N}\sum_{i=1}^{k}\sum_{j=1}^{n_{i}}K^{\left(i\right)}=K. \end{array}$$

Therefore,

(A12) $$\lim_{N\to \infty }V\left[\frac{1}{\sqrt{N}}\sum_{i=1}^{k}\sum_{j=1}^{n_{i}}\Psi_{\theta_{g}}\left(y_{ij}\right)\right]=K.$$

Finally, the asymptotic normality will be proved using the central limit theorem for the independent but not identical random variables using assumption (A5). A bound can be shown following Section 2.7 of [49] or Section 5 of [50] (also see [42]).

## References

[1] Fisher R.A. (1918). The correlation between relatives on the supposition of Mendelian inheritance. Philos. Trans. R. Soc. Edinb..

[2] Gelman A. (2005). Analysis of variance—Why it is more important than ever. Ann. Stat..

[3] Büning H. (1997). Robust analysis of variance. J. Appl. Stat..

[4] Armstrong R.A., Eperjesi F., Gilmartin B. (2002). The application of analysis of variance (ANOVA) to different experimental designs in optometry. Ophthalmic Physiol. Opt..

[5] Kohr R.L., Games P.A. (1974). Robustness of the analysis of variance, the Welch procedure and a Box procedure to heterogeneous variances. J. Exp. Educ..

[6] Gervini D., Yohai V.J. (1998). Robust estimation of variance components. Can. J. Stat..

[7] Fan W., Hancock G.R. (2012). Robust means modeling: An alternative for hypothesis testing of independent means under variance heterogeneity and nonnormality. J. Educ. Behav. Stat..

[8] Bertaccini B., Varriale R. (2007). Robust analysis of variance: An approach based on the forward search. Comput. Stat. Data Anal..

[9] Pearson E.S. (1931). The Analysis of variance in cases of non-normal variation. Biometrika.

[10] Büning H. (2000). Robustness and power of parametric, nonparametric, robustified and adaptive tests—the multi-sample location problem. Stat. Pap..

[11] Agostinelli C., Markatou M. (2001). Test of Hypotheses based on the weighted likelihood methodology. Stat. Sin..

[12] Tukey J.W. (1962). The future of data analysis. Ann. Math. Stat..

[13] Huber P.J. (1964). Robust estimation of a location parameter. Ann. Math. Stat..

[14] Andrews D.F., Bickel P.J., Hampel F.R., Huber P.J., Rogers W.H., Tukey J.W. (1972). Robust Estimation of Location: Survey and Advances.

[15] Hampel F.R. (1974). The influence curve and its role in robust estimation. J. Am. Stat. Assoc..

[16] Birch J.B., Myers R.H. (1982). Robust analysis of covariance. Biometrics.

[17] Tan W., Tabatabai M. (1985). Some robust ANOVA procedures under heteroscedasticity and nonnormality. Commun. Stat. -Simul. Comput..

[18] Schrader R.M., Hettmansperger T.P. (1980). Robust analysis of variance based upon a likelihood ratio criterion. Biometrika.

[19] Wilcox R.R., Charlin V.L., Thompson K.L. (1986). New monte carlo results on the robustness of the ANOVA F, W and *F*^*^ statistics. Commun. Stat. Comput..

[20] Brown M.B., Forsythe A.B. (1974). The small sample behavior of some statistics which test the equality of several means. Technometrics.

[21] Babu G.J., Padmanabhan A., Puri M.L. (1999). Robust one-way ANOVA under possibly non-regular conditions. Biom. J. J. Math. Methods Biosci..

[22] Kulinskaya E., Dollinger M.B. (2007). Robust weighted one-way ANOVA: Improved approximation and efficiency. J. Stat. Plan. Inference.

[23] Brunner E., Puri M.L. (2001). Nonparametric methods in factorial designs. Stat. Pap..

[24] Shuster J.J. (2005). Diagnostics for assumptions in moderate to large simple clinical trials: Do they really help?. Stat. Med..

[25] Huber P.J. (1981). Robust Statistics.

[26] Hampel F.R., Ronchetti E.M., Rousseeuw P.J., Stahel W.A. (1986). Robust Statistics: The Approach Based on Influence Functions.

[27] Heritier S., Cantoni E., Copt S., Victoria-Feser M.P. (2009). Robust Methods in Biostatistics.

[28] Farcomeni A., Ventura L. (2012). An overview of robust methods in medical research. Stat. Methods Med. Res..

[29] Dorph-Petersen K.A., Pierri J.N., Perel J.M., Sun Z., Sampson A.R., Lewis D.A. (2005). The influence of chronic exposure to antipsychotic medications on brain size before and after tissue fixation: A comparison of Haloperidol and Olanzapine in Macaque monkeys. Neuropsychopharmacology.

[30] Hosking S.M., Brennan-Olsen S.L., Beauchamp A., Buchbinder R., Williams L.J., Pasco J.A. (2018). Health literacy in a population-based sample of Australian women: A cross-sectional profile of the Geelong Osteoporosis Study. BMC Public Health.

[31] Pavel M.S., Chakrabarty S., Gow J. (2016). Cost of illness for outpatients attending public and private hospitals in Bangladesh. Int. J. Equity Health.

[32] Mititelu M., Stanciu G., Drǎgǎnescu D., Ioniţǎ A.C., Neacşu S.M., Dinu M., Stefan-van Staden R.I., Moroşan E. (2022). Mussel shells, a valuable calcium resource for the pharmaceutical industry. Mar. Drugs.

[33] Kishore K., Jaswal V., Mahajan R. (2022). The challenges of interpreting ANOVA by dermatologists. Indian Dermatol. Online J..

[34] Basu A., Harris I.R., Hjort N.L., Jones M.C. (1998). Robust and efficient estimation by minimising a density power divergence. Biometrika.

[35] Basu A., Ghosh A., Mandal A., Martin N., Pardo L. (2018). Robust Wald-type test in GLM with random design based on minimum density power divergence estimators. arXiv.

[36] Basu A., Mandal A., Martin N., Pardo L. (2015). Robust tests for the equality of two normal means based on the density power divergence. Metrika.

[37] Basu A., Shioya H., Park C. (2011). Statistical Inference: The Minimum Distance Approach.

[38] Pardo L. (2018). Statistical Inference Based on Divergence Measures.

[39] Fujisawa H., Eguchi S. (2008). Robust parameter estimation with a small bias against heavy contamination. J. Multivar. Anal..

[40] Ghosh A., Basu A. (2013). Robust estimation for independent non-homogeneous observations using density power divergence with applications to linear regression. Electron. J. Stat..

[41] Ibragimov I.A., Has’minskii R.Z. (1981). Statistical Estimation: Asymptotic Theory.

[42] Shih J.H., Konno Y., Chang Y.T., Emura T. (2022). Copula-based estimation methods for a common mean vector for bivariate meta-analyses. Symmetry.

[43] Shao J. (2003). Mathematical Statistics.

[44] Warwick J., Jones M. (2005). Choosing a robustness tuning parameter. J. Statist. Comput. Simul..

[45] Basu A., Ghosh A., Mandal A., Martin N., Pardo L. (2017). A Wald-type test statistic for testing linear hypothesis in logistic regression models based on minimum density power divergence estimator. Electron. J. Stat..

[46] Kałwak K., Porwolik J., Mielcarek M., Gorczyńska E., Owoc-Lempach J., Ussowicz M., Dyla A., Musiał J., Paździor D., Turkiewicz D. (2010). Higher CD34+ and CD3+ cell doses in the graft promote long-term survival, and have no impact on the incidence of severe acute or chronic graft-versus-host disease after in vivo T cell-depleted unrelated donor hematopoietic stem cell transplantation in children. Biol. Blood Marrow Transplant..

[47] Liu T., Fan W., Wu C. (2019). A hybrid machine learning approach to cerebral stroke prediction based on imbalanced medical dataset. Artif. Intell. Med..

[48] Dobler D., Pauly M. (2020). Factorial analyses of treatment effects under independent right-censoring. Stat. Methods Med. Res..

[49] Lehmann E.L. (1999). Elements of Large-Sample Theory.

[50] Ferguson T.S. (1996). A Course in Large Sample Theory.

