# Supplementary material for: Testing Equality of Multiple Population Means under Contaminated Normal Model Using the Density Power Divergence
Source: Entropy (Basel). 2022 Aug 25;24(9):1189. doi: 10.3390/e24091189 (PMC9497527; doi:10.3390/e24091189)
Supplement: Supplementary file 1 [file entropy-24-01189-s001.zip › entropy-1864859-supplementary.pdf]

Supplementary Materials: Testing Equality of Multiple Population Means under Contaminated Normal Model Using the Density Power Divergence

Jagannath Das [1], Beste Hamiye Beyaztas [2], Maxwell Kwesi Mac-Ocloo [1], Arunabha Majumdar [3] and Abhijit Mandal [1,*]

Supplementary Materials

A. Some Integrals

The following integrals are used in the DPD measure $d_\gamma(f_\theta, g)$ and simplify the J and K matrices at the model when $g = f_\theta$. As the integrals are over the entire real line, we will omit the subscripts from y_{ij} and call it y for simplicity. However, it should be noted that the mean function associated with y is μ_i.

$$\begin{aligned}\int_y f_\theta^{1+\gamma}(y)dy &= \left(\frac{1}{\sqrt{2\pi}\sigma}\right)^{1+\gamma}\int_y \exp\left\{-\frac{1+\gamma}{2\sigma^2}(y-\mu_i)^2\right\}\\ &= \left(\frac{1}{\sqrt{2\pi}\sigma}\right)^{1+\gamma}\frac{\sqrt{2\pi}\sigma}{\sqrt{1+\gamma}}\left[\frac{\sqrt{1+\gamma}}{\sqrt{2\pi}\sigma}\int_y \exp\left\{-\frac{1+\gamma}{2\sigma^2}(y-\mu_i)^2\right\}\right]\\ &= (2\pi)^{-\frac{\gamma}{2}}\sigma^{-\gamma}(1+\gamma)^{-\frac{1}{2}}.\end{aligned} \tag{A.1}$$

$$\int_y (y-\mu_i) f_\theta^{1+\gamma}(y)dy = \int_y (y-\mu_i)^3 f_\theta^{1+\gamma}(y)dy = 0. \tag{A.2}$$

$$\begin{aligned}\int_y (y-\mu_i)^2 f_\theta^{1+\gamma}(y)dy &= \left(\frac{1}{\sqrt{2\pi}\sigma}\right)^{1+\gamma}\int_y (y-\mu_i)^2\exp\left\{-\frac{1+\gamma}{2\sigma^2}(y-\mu_i)^2\right\}\\ &= \left(\frac{1}{\sqrt{2\pi}\sigma}\right)^{1+\gamma}\frac{\sqrt{2\pi}\sigma}{\sqrt{1+\gamma}}\left[\frac{\sqrt{1+\gamma}}{\sqrt{2\pi}\sigma}\int_y (y-\mu_i)^2\exp\left\{-\frac{1+\gamma}{2\sigma^2}(y-\mu_i)^2\right\}\right]\\ &= (2\pi)^{-\frac{\gamma}{2}}\sigma^{-\gamma}(1+\gamma)^{-\frac{1}{2}}\frac{\sigma^2}{1+\gamma}\\ &= (2\pi)^{-\frac{\gamma}{2}}\sigma^{-\gamma+2}(1+\gamma)^{-\frac{3}{2}}.\end{aligned} \tag{A.3}$$

$$\begin{aligned}\int_y (y-\mu_i)^4 f_\theta^{1+\gamma}(y)dy &= \left(\frac{1}{\sqrt{2\pi}\sigma}\right)^{1+\gamma}\int_y (y-\mu_i)^4\exp\left\{-\frac{1+\gamma}{2\sigma^2}(y-\mu_i)^2\right\}\\ &= \left(\frac{1}{\sqrt{2\pi}\sigma}\right)^{1+\gamma}\frac{\sqrt{2\pi}\sigma}{\sqrt{1+\gamma}}\left[\frac{\sqrt{1+\gamma}}{\sqrt{2\pi}\sigma}\int_y (y-\mu_i)^4\exp\left\{-\frac{1+\gamma}{2\sigma^2}(y-\mu_i)^2\right\}\right]\\ &= (2\pi)^{-\frac{\gamma}{2}}\sigma^{-\gamma}(1+\gamma)^{-\frac{1}{2}}\frac{3\sigma^4}{(1+\gamma)^2}\\ &= 3(2\pi)^{-\frac{\gamma}{2}}\sigma^{-\gamma+4}(1+\gamma)^{-\frac{5}{2}}.\end{aligned} \tag{A.4}$$

B. Estimating Equations

From Equation (4) of the main paper, we get

$$\begin{aligned} &\frac{\partial}{\partial \mu_i}\widehat{d}_\gamma(f_\theta, g) = 0 \\ &\implies \frac{\partial}{\partial \mu_i}\sum_{j=1}^{n_i}\exp\Big\{-\frac{\gamma}{2\sigma^2}(y_{ij}-\mu_i)^2\Big\} = 0 \\ &\implies \sum_{j=1}^{n_i}\exp\Big\{-\frac{\gamma}{2\sigma^2}(y_{ij}-\mu_i)^2\Big\}\frac{\partial}{\partial \mu_i}(y_{ij}-\mu_i)^2 = 0 \\ &\implies \sum_{j=1}^{n_i}(y_{ij}-\mu_i)\exp\Big\{-\frac{\gamma}{2\sigma^2}(y_{ij}-\mu_i)^2\Big\} = 0, \end{aligned} \tag{B.1}$$

and

$$\begin{aligned} &\frac{\partial}{\partial \sigma^2}\widehat{d}_\gamma(f_\theta, g) = 0 \\ &\implies \frac{\partial}{\partial \sigma^2}\left\{\sigma^{-\gamma}\left[1 - \frac{(1+\gamma)^{3/2}}{N\gamma}\sum_{i=1}^{k}\sum_{j=1}^{n_i}\exp\Big\{-\frac{\gamma}{2\sigma^2}(y_{ij}-\mu_i)^2\Big\}\right]\right\} = 0 \\ &\implies \left[\frac{\partial}{\partial \sigma^2}\sigma^{-\gamma}\right]\left[1 - \frac{(1+\gamma)^{3/2}}{N\gamma}\sum_{i=1}^{k}\sum_{j=1}^{n_i}\exp\Big\{-\frac{\gamma}{2\sigma^2}(y_{ij}-\mu_i)^2\Big\}\right] \\ &\qquad - \frac{(1+\gamma)^{3/2}\sigma^{-\gamma}}{N\gamma}\sum_{i=1}^{k}\sum_{j=1}^{n_i}\frac{\partial}{\partial \sigma^2}\exp\Big\{-\frac{\gamma}{2\sigma^2}(y_{ij}-\mu_i)^2\Big\} = 0 \\ &\implies -\frac{\gamma}{2}\sigma^{-\gamma-2}\left[1 - \frac{(1+\gamma)^{3/2}}{N\gamma}\sum_{i=1}^{k}\sum_{j=1}^{n_i}\exp\Big\{-\frac{\gamma}{2\sigma^2}(y_{ij}-\mu_i)^2\Big\}\right] \\ &\qquad - \frac{\gamma(1+\gamma)^{3/2}\sigma^{-\gamma-4}}{2N\gamma}\sum_{i=1}^{k}\sum_{j=1}^{n_i}(y_{ij}-\mu_i)^2\exp\Big\{-\frac{\gamma}{2\sigma^2}(y_{ij}-\mu_i)^2\Big\} = 0 \\ &\implies 1 - \frac{(1+\gamma)^{3/2}}{N\gamma}\sum_{i=1}^{k}\sum_{j=1}^{n_i}\exp\Big\{-\frac{\gamma}{2\sigma^2}(y_{ij}-\mu_i)^2\Big\} \\ &\qquad + \frac{(1+\gamma)^{3/2}}{N\gamma\sigma^2}\sum_{i=1}^{k}\sum_{j=1}^{n_i}(y_{ij}-\mu_i)^2\exp\Big\{-\frac{\gamma}{2\sigma^2}(y_{ij}-\mu_i)^2\Big\} = 0. \end{aligned} \tag{B.2}$$

Thus, the estimating equations of θ are obtained from equation $\frac{\partial}{\partial\theta}\widehat{d}_\gamma(f_\theta, g) = 0$ and they are simplified as

$$\begin{aligned} &\sum_{j=1}^{n_i}(y_{ij}-\mu_i)\exp\Big\{-\frac{\gamma}{2\sigma^2}(y_{ij}-\mu_i)^2\Big\} = 0 \ \text{ for } i = 1, 2, \cdots, k, \\ &1 - \frac{(1+\gamma)^{3/2}}{N\gamma}\sum_{i=1}^{k}\sum_{j=1}^{n_i}\exp\Big\{-\frac{\gamma}{2\sigma^2}(y_{ij}-\mu_i)^2\Big\} \\ &\qquad + \frac{(1+\gamma)^{3/2}}{N\gamma\sigma^2}\sum_{i=1}^{k}\sum_{j=1}^{n_i}(y_{ij}-\mu_i)^2\exp\Big\{-\frac{\gamma}{2\sigma^2}(y_{ij}-\mu_i)^2\Big\} = 0. \end{aligned} \tag{B.3}$$

C. Score Functions

The probability density function is given by

$$f_\theta(y_{ij}) = \frac{1}{\sqrt{2\pi}\sigma}\exp\Big\{-\frac{1}{2\sigma^2}(y_{ij}-\mu_i)^2\Big\}, \tag{C.1}$$

Let us define the score function as

$$
\begin{aligned}
u_\theta(y_{ij}) &= \frac{\partial}{\partial\theta}\log f_\theta(y_{ij}) \\
&= \frac{\partial}{\partial\theta}\left[-\frac{1}{2}\log(2\pi) - \frac{1}{2}\log\sigma^2 - \frac{1}{2\sigma^2}(y_{ij}-\mu_i)^2\right].
\end{aligned}
\tag{C.2}
$$

We write

$$
u_\theta(y_{ij}) = (u_{\mu_1}(y_{ij}), u_{\mu_2}(y_{ij}), \cdots, u_{\mu_k}(y_{ij}), u_{\sigma^2}(y_{ij}))^T. \tag{C.3}
$$

For parameters μ_i and σ^2, the score functions are given by

$$
\begin{aligned}
u_{\mu_i}(y_{ij}) &= \frac{\partial}{\partial\mu_i}\log f_\theta(y_{ij}) \\
&= \frac{\partial}{\partial\mu_i}\left[-\frac{1}{2}\log(2\pi) - \frac{1}{2}\log\sigma^2 - \frac{1}{2\sigma^2}(y_{ij}-\mu_i)^2\right] \\
&= \frac{1}{\sigma^2}(y_{ij}-\mu_i),
\end{aligned}
\tag{C.4}
$$

$$
u_{\mu_r}(y_{ij}) = \frac{\partial}{\partial\mu_r}\log f_\theta(y_{ij}) = 0, \text{ for } r \neq i, \tag{C.5}
$$

$$
\begin{aligned}
u_{\sigma^2}(y_{ij}) &= \frac{\partial}{\partial\sigma^2}\log f_\theta(y_{ij}) \\
&= \frac{\partial}{\partial\sigma^2}\left[-\frac{1}{2}\log(2\pi) - \frac{1}{2}\log\sigma^2 - \frac{1}{2\sigma^2}(y_{ij}-\mu_i)^2\right] \\
&= -\frac{1}{2\sigma^2} + \frac{1}{2\sigma^4}(y_{ij}-\mu_i)^2.
\end{aligned}
\tag{C.6}
$$

D. J and K matrices at the Model

Note that, if the true distribution $g(y)$ is a member of the model family $f_\theta(y)$ for some $\theta \in \Theta$, then

$$
J^{(ij)} = \int_y u_\theta(y)u_\theta^T(y)f_\theta^{1+\gamma}(y)dy. \tag{D.1}
$$

In this case, the symmetric matrix $J^{(ij)}$ can be partitioned as

$$
J^{(ij)} = \begin{bmatrix}
J^{(ij)}_{\mu_1} & J^{(ij)}_{\mu_1,\mu_2} & J^{(ij)}_{\mu_1,\mu_3} & \cdots & J^{(ij)}_{\mu_1,\mu_k} & J^{(ij)}_{\mu_1,\sigma^2} \\
. & J^{(ij)}_{\mu_2} & J^{(ij)}_{\mu_2,\mu_3} & \cdots & J^{(ij)}_{\mu_2,\mu_k} & J^{(ij)}_{\mu_2,\sigma^2} \\
\vdots & \vdots & \vdots & \vdots & \vdots & \vdots \\
. & . & . & \cdots & J^{(ij)}_{\mu_k} & J^{(ij)}_{\mu_k,\sigma^2} \\
. & . & . & \cdots & . & J^{(ij)}_{\sigma^2}
\end{bmatrix}, \tag{D.2}
$$

and in Appendix F, it is shown that

$$
\begin{aligned}
J^{(ij)}_{\mu_i} &= (2\pi)^{-\frac{\gamma}{2}}\sigma^{-\gamma-2}(1+\gamma)^{-\frac{3}{2}}, \\
J^{(ij)}_{\mu_r} &= 0, \text{ for } r \neq i, \\
J^{(ij)}_{\mu_r,\mu_s} &= 0, \text{ for } r \neq s, \\
J^{(ij)}_{\sigma^2} &= \frac{1}{4}(2\pi)^{-\frac{\gamma}{2}}\sigma^{-\gamma-4}(1+\gamma)^{-\frac{5}{2}}(2+\gamma^2), \\
J^{(i)}_{\mu_i,\,\sigma^2} &= 0.
\end{aligned}
\tag{D.3}
$$

The $J^{(ij)}$ matrix simplifies to

$$J^{(ij)} = (2\pi)^{-\frac{\gamma}{2}}\sigma^{-\gamma-2}(1+\gamma)^{-\frac{3}{2}}\begin{bmatrix} D_i & 0_k \\ 0_k^T & \frac{(2+\gamma^2)}{4\sigma^2(1+\gamma)} \end{bmatrix}, \tag{D.4}$$

where D_i is a $k \times k$ dimensional matrix with (i,i)-th diagonal element 1 and 0 otherwise. Therefore,

$$J = \lim_{N\to\infty}\frac{1}{N}\sum_{i=1}^{k}\sum_{j=1}^{n_i} J^{(ij)} = (2\pi)^{-\frac{\gamma}{2}}\sigma^{-\gamma-2}(1+\gamma)^{-\frac{3}{2}}\lim_{N\to\infty}\begin{bmatrix} S & 0_k \\ 0_k^T & \frac{(2+\gamma^2)}{4\sigma^2(1+\gamma)} \end{bmatrix}, \tag{D.5}$$

where S is a $k \times k$ dimensional diagonal matrix with i-th diagonal element n_i/N. Similarly, $\xi^{(ij)}$ can be partitioned as $\xi^{(ij)} = \left(\xi^{(ij)}_{\mu_1}, \xi^{(ij)}_{\mu_2}, \cdots, \xi^{(ij)}_{\mu_k}, \xi^{(ij)}_{\sigma^2}\right)^T$, and in Appendix E, it is shown that

$$\xi^{(ij)}_{\mu_i} = 0, \text{ and } \xi^{(ij)}_{\sigma^2} = -\frac{\gamma}{2}(2\pi)^{-\frac{\gamma}{2}}\sigma^{-\gamma-2}(1+\gamma)^{-\frac{3}{2}}. \tag{D.6}$$

Note that if we write the matrix $J^{(ij)}$ as a function of γ, i.e., $J^{(ij)} \equiv J^{(ij)}(\gamma)$, then we have

$$K^{(ij)} = J^{(ij)}(2\gamma) - \xi^{(ij)}\xi^{(ij)T}. \tag{D.7}$$

So K can be written as

$$K = \lim_{N\to\infty}\frac{1}{N}\sum_{i=1}^{k}\sum_{j=1}^{n_i} J^{(ij)}(2\gamma) - \xi^{(ij)}\xi^{(ij)T}. \tag{D.8}$$

Using Equations (D.5) and (D.6), we get from Equation (D.8)

$$K = (2\pi)^{-\gamma}\sigma^{-2\gamma-2}(1+2\gamma)^{-\frac{3}{2}}\lim_{N\to\infty}\begin{bmatrix} S & 0_k \\ 0_k^T & \nu \end{bmatrix}, \tag{D.9}$$

where

$$\begin{aligned} \nu &= \frac{(1+2\gamma^2)}{2\sigma^2(1+2\gamma)} - \frac{\gamma^2}{4}(2\pi)^{-\gamma}\sigma^{-2\gamma-4}(1+\gamma)^{-3}\frac{1}{(2\pi)^{-\gamma}\sigma^{-2\gamma-2}(1+2\gamma)^{-\frac{3}{2}}} \\ &= \frac{(1+2\gamma^2)}{2\sigma^2(1+2\gamma)} - \frac{\gamma^2(1+2\gamma)^{\frac{3}{2}}}{4\sigma^2(1+\gamma)^3}. \end{aligned} \tag{D.10}$$

E. Vector $\xi^{(ij)}$ at Model

From Equations (D.1) and (C.4), we get

$$\begin{aligned} \xi^{(ij)}_{\mu_i} &= \int_y u_{\mu_i}(y) f_\theta^{1+\gamma}(y)dy \\ &= \int_y \frac{1}{\sigma^2}(y-\mu_i) f_\theta^{1+\gamma}(y)dy \\ &= 0, \quad \text{from (A.2)}. \end{aligned} \tag{E.1}$$

From Equations (D.1) and (C.6), we get

$$\begin{aligned}
\xi^{(ij)}_{\sigma^2} &= \int_y u_{\sigma^2}(y) f_\theta^{1+\gamma}(y)dy \\
&= \int_y \frac{1}{\sigma^2}(y-\mu_i) f_\theta^{1+\gamma}(y)dy \ \text{ should not be here} \\
&= \int_y \left[-\frac{1}{2\sigma^2} + \frac{1}{2\sigma^4}(y-\mu_i)^2\right] f_\theta^{1+\gamma}(y)dy \\
&= -\frac{1}{2\sigma^2}(2\pi)^{-\frac{\gamma}{2}}\sigma^{-\gamma}(1+\gamma)^{-\frac{1}{2}} \ \text{ from (A.1)} \\
&\quad + \frac{1}{2\sigma^4}(2\pi)^{-\frac{\gamma}{2}}\sigma^{-\gamma+2}(1+\gamma)^{-\frac{3}{2}} \ \text{ from (A.3)} \\
&= -\frac{\gamma}{2}(2\pi)^{-\frac{\gamma}{2}}\sigma^{-\gamma-2}(1+\gamma)^{-\frac{3}{2}}.
\end{aligned} \tag{E.2}$$

F. Matrix $J^{(ij)}$ at Model

From Equations (D.1) and (C.4), we get

$$\begin{aligned}
J^{(ij)}_{\mu_i} &= \int_y u^2_{\mu_i}(y) f_\theta^{1+\gamma}(y)dy \\
&= \int_y \frac{1}{\sigma^4}(y-\mu_i)^2 f_\theta^{1+\gamma}(y)dy \\
&= \frac{1}{\sigma^4}(2\pi)^{-\frac{\gamma}{2}}\sigma^{-\gamma+2}(1+\gamma)^{-\frac{3}{2}} \ \text{ from (A.3)} \\
&= (2\pi)^{-\frac{\gamma}{2}}\sigma^{-\gamma-2}(1+\gamma)^{-\frac{3}{2}}.
\end{aligned} \tag{F.1}$$

$$\begin{aligned}
J^{(ij)}_{\mu_r} &= 0, \ \text{for } r \neq i, \\
J^{(ij)}_{\mu_r,\mu_s} &= 0, \ \text{for } r \neq s,
\end{aligned} \tag{F.2}$$

From Equations (D.1) and (C.6), we get

$$\begin{aligned}
J^{(ij)}_{\sigma^2} &= \int_y u^2_{\sigma^2}(y) f_\theta^{1+\gamma}(y)dy \\
&= \int_y \left[-\frac{1}{2\sigma^2} + \frac{1}{2\sigma^4}(y-\mu_i)^2\right]^2 f_\theta^{1+\gamma}(y)dy \\
&= \frac{1}{4\sigma^4}(2\pi)^{-\frac{\gamma}{2}}\sigma^{-\gamma}(1+\gamma)^{-\frac{1}{2}} \ \text{ from (A.1)} \\
&\quad - \frac{1}{2\sigma^6}(2\pi)^{-\frac{\gamma}{2}}\sigma^{-\gamma+2}(1+\gamma)^{-\frac{3}{2}} \ \text{ from (A.3)} \\
&\quad + \frac{1}{4\sigma^8}3(2\pi)^{-\frac{\gamma}{2}}\sigma^{-\gamma+4}(1+\gamma)^{-\frac{5}{2}} \ \text{ from (A.4)} \\
&= \frac{1}{4}(2\pi)^{-\frac{\gamma}{2}}\sigma^{-\gamma-4}(1+\gamma)^{-\frac{5}{2}}\left[(1+\gamma)^2 - 2(1+\gamma) + 3\right] \\
&= \frac{1}{4}(2\pi)^{-\frac{\gamma}{2}}\sigma^{-\gamma-4}(1+\gamma)^{-\frac{5}{2}}(2+\gamma^2)
\end{aligned} \tag{F.3}$$

From Equations (D.1) and (C.6), we get

$$\begin{aligned}
J^{(ij)}_{\mu_i,\,\sigma^2} &= \int_y u_{\mu_i}(y) u_{\sigma^2}(y) f_\theta^{1+\gamma}(y)dy \\
&= \int_y \frac{1}{\sigma^2}(y-\mu_i)\left[-\frac{1}{2\sigma^2} + \frac{1}{2\sigma^4}(y-\mu_i)^2\right] f_\theta^{1+\gamma}(y)dy \\
&= 0 \ \text{ from (A.2)}.
\end{aligned} \tag{F.4}$$

G. Test Statistics

From Equation (D.5), we get

$$J = \lim_{N\to\infty} \frac{1}{N} \sum_{i=1}^{k} \sum_{j=1}^{n_i} J^{(ij)} = (2\pi)^{-\frac{\gamma}{2}} \sigma^{-\gamma-2} (1+\gamma)^{-\frac{3}{2}} \lim_{N\to\infty} \begin{bmatrix} S & 0_k \\ 0_k^T & \frac{(2+\gamma^2)}{4\sigma^2(1+\gamma)} \end{bmatrix} = \lim_{N\to\infty} J_N, \quad \text{(G.1)}$$

where S is a $k \times k$ dimensional diagonal matrix with i-th diagonal element n_i/N. Using the inverse of a block matrix, we have

$$J_N^{-1} = d \begin{bmatrix} S^{-1} & 0_k \\ 0_k^T & \frac{4\sigma^2(1+\gamma)}{(2+\gamma^2)} \end{bmatrix}, \quad \text{(G.2)}$$

where $d = (2\pi)^{\frac{\gamma}{2}} \sigma^{\gamma+2} (1+\gamma)^{\frac{3}{2}}$. From the definition of M in Section **??**, we have

$$M = \begin{bmatrix} M_\mu \\ 0_{k-1}^T \end{bmatrix}. \quad \text{(G.3)}$$

Now

$$M^T J_N^{-1} = d \begin{bmatrix} M_\mu^T S^{-1} & 0_{k-1} \end{bmatrix}. \quad \text{(G.4)}$$

From Equation (D.9), we have

$$K = h \lim_{N\to\infty} \begin{bmatrix} S & 0_k \\ 0_k^T & \nu \end{bmatrix}, \quad \text{(G.5)}$$

where $h = (2\pi)^{-\gamma} \sigma^{-2\gamma-2} (1+2\gamma)^{-\frac{3}{2}}$. We write

$$K = \lim_{N\to\infty} K_N.$$

Then

$$\begin{aligned} M^T J_N^{-1} K_N M J_N^{-1} M &= d^2 h \begin{bmatrix} M_\mu^T S^{-1} & 0_{k-1} \end{bmatrix} \begin{bmatrix} S & 0_k \\ 0_k^T & \nu \end{bmatrix} \begin{bmatrix} M_\mu^T S^{-1} & 0_{k-1} \end{bmatrix}^T \\ &= d^2 h \begin{bmatrix} M_\mu^T & 0_{k-1} \end{bmatrix} \begin{bmatrix} M_\mu^T S^{-1} & 0_{k-1} \end{bmatrix}^T \\ &= d^2 h M_\mu^T S^{-1} M_\mu, \end{aligned} \quad \text{(G.6)}$$

where

$$\begin{aligned} d^2 h &= (2\pi)^{\gamma} \sigma^{2\gamma+4} (1+\gamma)^3 \times (2\pi)^{-\gamma} \sigma^{-2\gamma-2} (1+2\gamma)^{-\frac{3}{2}} \\ &= \sigma^2 (1+\gamma)^3 (1+2\gamma)^{-\frac{3}{2}}. \end{aligned} \quad \text{(G.7)}$$

Combining Equations (G.6) and (G.7) , we get

$$M^T J_N^{-1} K_N M J_N^{-1} M = \sigma^2 (1+\gamma)^3 (1+2\gamma)^{-\frac{3}{2}} M_\mu^T S^{-1} M_\mu. \quad \text{(G.8)}$$

H. Matrix Σ_μ

We have $\Sigma_\theta = J^{-1} K J^{-1}$, where

$$J = (2\pi)^{-\frac{\gamma}{2}} \sigma^{-\gamma-2} (1+\gamma)^{-\frac{3}{2}} \lim_{N\to\infty} \begin{bmatrix} S & 0_k \\ 0_k^T & \frac{(2+\gamma^2)}{4\sigma^2(1+\gamma)} \end{bmatrix} = d(\gamma) \lim_{N\to\infty} J_N, \quad \text{(H.1)}$$

$$K = (2\pi)^{-\gamma} \sigma^{-2\gamma-2} (1+2\gamma)^{-\frac{3}{2}} \lim_{N\to\infty} \begin{bmatrix} S & 0_k \\ 0_k^T & \nu \end{bmatrix} = d(2\gamma) \lim_{N\to\infty} K_N, \quad \text{(H.2)}$$

and $d(\gamma) = (2\pi)^{-\frac{\gamma}{2}} \sigma^{-\gamma-2} (1+\gamma)^{-\frac{3}{2}}$. Now,

$$J_N^{-1} = \begin{bmatrix} S^{-1} & 0_k \\ 0_k^T & \frac{1}{\eta} \end{bmatrix}, \tag{H.3}$$

where $\eta = \frac{(2+\gamma^2)}{4\sigma^2(1+\gamma)}$. Therefore,

$$\begin{aligned} J^{-1}KJ^{-1} &= \frac{d(2\gamma)}{d^2(\gamma)} \lim_{N\to\infty} J_N^{-1} K_N J_N^{-1} \\ &= \frac{d(2\gamma)}{d^2(\gamma)} \lim_{N\to\infty} \begin{bmatrix} I_K & 0_k \\ 0_k^T & \frac{\nu}{\eta} \end{bmatrix} \begin{bmatrix} S^{-1} & 0_k \\ 0_k^T & \frac{1}{\eta} \end{bmatrix} \\ &= \frac{(1+\gamma)^3 \sigma^2}{(1+2\gamma)^{\frac{3}{2}}} \lim_{N\to\infty} \begin{bmatrix} S^{-1} & 0_k \\ 0_k^T & \frac{\nu}{\eta^2} \end{bmatrix}. \end{aligned} \tag{H.4}$$

Thus, the covariance matrix of $\sqrt{N}\widehat{\mu}$ is $\Sigma_\mu = \frac{(1+\gamma)^3\sigma^2}{(1+2\gamma)^{\frac{3}{2}}} \lim_{N\to\infty} S^{-1}$. Similarly, the variance of $\sqrt{N}\widehat{\sigma}$ is given by $\sigma_\gamma = \frac{(1+\gamma)^3\sigma^2}{(1+2\gamma)^{\frac{3}{2}}} \frac{\nu}{\eta^2} = \frac{\sigma^4(1+\gamma)^2}{(2+\gamma^2)^2} \left\{ \frac{2(1+\gamma)^3(1+2\gamma^2)}{(1+2\gamma)^{\frac{5}{2}}} - \gamma^2 \right\}$. Finally, $\widehat{\mu}$ and $\widehat{\sigma}$ are asymptotically independent.
